# Palladium Membrane Applications in Hydrogen Energy and Hydrogen-Related Processes

**DOI:** 10.3390/polym17060743

**Published:** 2025-03-12

**Authors:** Dmitry A. Alentiev, Maxim V. Bermeshev, Alexey V. Volkov, Inna V. Petrova, Andrey B. Yaroslavtsev

**Affiliations:** 1 A.V. Topchiev Institute of Petrochemical Synthesis of Russian Academy of Sciences, 29 Leninskiy Prospekt, 119991 Moscow, Russia; bmv@ips.ac.ru (M.V.B.); avolkov@ips.ac.ru (A.V.V.); ivpetrova@ips.ac.ru (I.V.P.); 2 N.S. Kurnakov Institute of General and Inorganic Chemistry of Russian Academy of Sciences, 31 Leninskiy Prospekt, 119991 Moscow, Russia

**Keywords:** palladium membranes, polymer–palladium composites, hydrogen purification, membrane reactors, water purification

## Abstract

In recent years, increased attention has been paid to environmental issues and, in connection with this, to the development of hydrogen energy. In turn, this requires the large-scale production of ultra pure hydrogen. Currently, most hydrogen is obtained by converting natural gas and coal. In this regard, the issue of the deep purification of hydrogen for use in fuel cells is very relevant. The deep purification of hydrogen is also necessary for some other areas, including microelectronics. Only palladium membranes can provide the required degree of purification. In addition, the use of membrane catalysis is very relevant for the widely demanded processes of hydrogenation and dehydrogenation, for which reactors with palladium membranes are used. This process is also successfully used for the single-stage production of high-purity hydrogen. Polymeric palladium-containing membranes are also used to purify hydrogen and to remove various pollutants from water, including organochlorine products, nitrates, and a number of other substances.

## 1. Introduction

Noble metals have been a focal point of researchers for a long time due to their unique properties. Despite their low chemical activity, which allows them to be used in processes occurring in fairly aggressive environments, they exhibit pronounced sorption properties. With the benefit of rather balanced sorption and desorption capacities for various molecules and ions, they are characterized by high catalytic activity and are commonly used as catalysts for a wide range of chemical processes [1,2,3,4]. Platinum-based materials are primarily used for this purpose. Other noble metals display equally interesting properties, such as palladium, which is an important metal in multiple catalytic applications [5,6,7,8,9,10]. Palladium has secured its niche in hydrogen transfer applications. Although palladium is not the only metal with a highly selective hydrogen transfer capacity, and it is not the fastest hydrogen transporter [11], it is far superior to its competitors when it comes to stability, which has supported its extensive application in a variety of processes. For this reason, many researchers consider palladium-based materials to be highly relevant for hydrogen-related processes.

Over time, the application of palladium-based materials has become much more diverse. Gryaznov’s discovery of the coupling of hydrogen absorption–desorption reactions on a palladium-containing membrane marked a significant milestone [12,13]. Research in this area progressed rapidly and translated into a new scientific discipline of membrane catalysis, in which field several well-known scientific groups are active and international conferences are held regularly [14,15,16,17,18].

Public concern over environmental issues has stimulated research in the field of hydrogen energy, where palladium-based materials may not only be used for hydrogen extraction and purification but also for H_2_ generation using membrane catalysis, which combines hydrogen production with its deep purification [19,20,21]. Palladium can also be used as an effective catalyst in fuel cells. Although, in this respect, palladium is somewhat inferior to platinum, palladium-based alloys present a promising area for research [22,23,24]. Another promising area is the application of membranes with a palladium coating, which is based on palladium’s ability to accumulate hydrogen and catalyze processes with its participation due to the dissociative sorption of hydrogen, which passes into the most active atomic state [25,26]. This, in particular, allows the use of such membranes in chemical processes, reducing a number of substances that pose a threat to various production processes or to individuals [27,28,29]. Of course, this short list cannot cover all the possible palladium applications, but it provides a fairly good view of the features of palladium and palladium-based alloy membranes that make them applicable in a variety of commercial processes.

In this respect, the main objective of this publication is to provide a brief overview of hydrogen production and purification methods using palladium-containing membranes. There are various types of membranes based on palladium, including membranes based on its alloys and polymer membranes modified with palladium, as well as various processes involving them, including hydrogenation, dehydrogenation, and hydrogen production using membrane catalysis. The use of palladium-containing polymer membranes in fuel cells and water purification processes is also considered in this review.

## 2. Key Hydrogen Production Methods

In recent years, the world’s focus on environmental issues has seriously increased in response to the active utilization of fossil fuels by industries, transport, and human activities [30,31]. According to the global warming theory, climate change is caused by the emission of anthropogenic greenhouse gases [32,33,34,35]. As most emissions come from power generation for grids, industrial operations, and vehicles, the major trends in today’s energy industry are saving energy and developing renewable energy sources [36] for the decarbonization of the economy [37,38,39]. Additionally, technologies often advance on the back of greater power demand, which makes the development of environmentally friendly renewable energy sources even more critical. Along with solar panels and wind turbines, hydrogen energy is also gaining traction. Hydrogen energy can be used for power supply in remote, isolated areas, where grid power supply is not available, as a source of backup power supply and for powering and charging vehicles. One of the major applications of hydrogen relates to energy storage, without which it is impossible to ensure a reliable power supply from solar, wind, and other renewable energy sources that are inherently unstable [40,41,42,43,44,45]. The demand for hydrogen is driven by its extremely high energy intensity and the sustainability of energy production during its oxidation. In addition, when using fuel cells, the efficiency of the oxidation process is very high and can exceed 70% [46,47,48].

However, on Earth, hydrogen exists only in a bound state. According to the International Energy Agency, the global hydrogen production volume in 2020 ranged from 90 to 115 million tons, and by 2050, it will have increased by 2–6 times [49,50]. More than half of the hydrogen currently produced is used for ammonia synthesis, with the remaining 41% used for methanol production and oil refining; by 2050, about 80% of hydrogen produced is expected to be utilized in power generation and as a fuel for transport [20,51,52].

### 2.1. Hydrogen Production from Natural Gas

**Figure 1:**
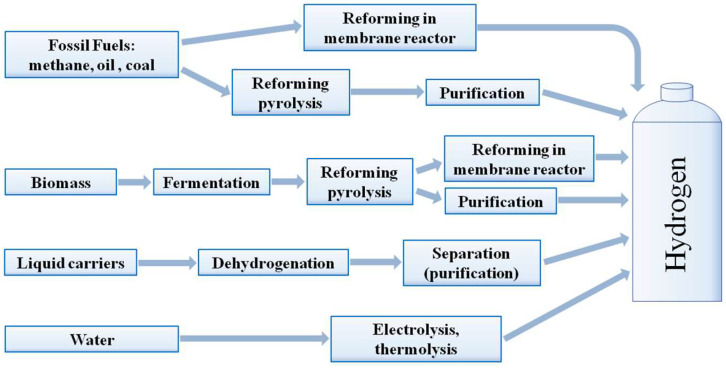
Key hydrogen production methods.

The key hydrogen production methods are shown in Figure 1. Currently, the main precursor for these processes is natural gas [20,53,54,55], and among its processing methods, steam reforming should be highlighted first, which involves the following reaction:

CH_4_ + H_2_O = CO + 3H_2_, ΔH° = 206 kJ/mol (1)

Its advantage is a high hydrogen yield. The endothermic nature of this reaction and sufficient stability of the methane molecule require relatively high energy input and allow the process to run only at sufficiently high temperatures, about 700–900 °C [56,57,58]. An obvious disadvantage is carbon monoxide being a major byproduct. It is toxic not only to humans but also to the catalysts of the most common low-temperature fuel cells [59,60]. To reduce the CO concentration and increase hydrogen recovery, the products are subjected to the water–gas shift reaction at the second stage:

CO + H_2_O = CO_2_ + H_2_, ΔH° = −41 kJ/mol (2)

As this process is exothermic, it runs at a lower temperature (200–400 °C). Combined, these processes can produce up to four volume units of hydrogen per one volume unit of methane.

Partial methane oxidation, which occurs according to Equation (3), is characterized by a theoretical yield of hydrogen that is two times smaller [61,62].

CH_4_ + 1/2O_2_ = CO + 2H_2_, ΔH° = −8.6 kJ/mol (3)

The advantage of this process is its exothermic nature, but a strong oxidizer and a high temperature decrease the selectivity, which is manifested as carbon depositions on the catalyst that deactivate the catalyst and create partial hydrogen oxidation [63,64]. An autothermal methane reforming process, which combines methane steam reforming with its partial oxidation, can be used for the optimization of the process’s energy consumption [65]. However, this does not change the reaction products composition, as the heat released at the methane partial oxidation stage is 20 times less compared to the heat required for the steam reforming. The high ratio of carbon monoxide (which is a catalytic poison for fuel cells) to hydrogen in the methane partial oxidation products indicates the benefit of their application in organic synthesis, including artificial fuels, methanol, etc. [61,62], while the extraction of clean hydrogen from them has less merit.

A process known as dry methane reforming is characterized by an even higher CO/hydrogen ratio in its products [66]:

CH_4_ + CO_2_ = 2CO + 2H_2_, ΔH° = 248 kJ/mol (4)

This process is considered promising only because it is possible to use it for CO_2_ recovery [37,67]. However, due to the high CO content in products, it should not be considered for hydrogen production.

From a carbon footprint reduction standpoint, the natural gas pyrolysis is of interest, because solid carbon is a single by-product according to the following reaction [68,69,70]:

CH_4_ = C + 2H_2_, ΔH° = 74.8 kJ/mol (5)

However, due to a high reaction temperature and natural gas contamination, the process generates a number of gaseous impurities, and the final product requires deep purification. Also, the generated carbon disposal is a serious issue, as it will accumulate much quicker than it can be consumed in the case of large-scale operations.

According to [71], less than a quarter of hydrogen produced is accounted for by coal gasification, although this process is the most harmful to the environment, and it is currently being phased out [72,73,74].

### 2.2. Liquid Hydrogen Carriers

Although the hydrogen energy intensity per unit mass is the highest among the other energy vectors, its volumetric energy intensity is very low. Therefore, the issue of hydrogen compression for transportation is very relevant. This process requires high energy consumption, and its mass fraction in cylinders is very low. From the energy costs perspective, it is even more challenging to liquefy hydrogen and keep it in a liquid state during transportation [75,76]. In this regard, significant attention is paid to the use of so-called liquid carriers—unsaturated, often aromatic compounds that easily bind hydrogen and then easily release it at elevated temperatures (Figure 1). They include toluene, cyclohexane, methylcyclohexane, decalin, and a number of heterocyclic compounds characterized by a lower dehydrogenation temperature [77,78,79,80,81,82]. The theoretical capacity of liquid carriers can exceed 8%. Liquid hydrogen carriers also include ammonia, the byproduct of whose decomposition is nitrogen, which does not need to be disposed of or transported as it can be easily extracted via liquefaction or distillation from the atmosphere. Ammonia can provide an even higher hydrogen storage density [83,84,85]; however, for its sufficient conversion, a high temperature is required, which affects energy efficiency and the economy of the process. Moreover, the products of this process contain ammonia, which, even in trace amounts, can poison proton-conducting membranes and affect their permeability, even if present in trace amounts. This makes it impossible to use the produced hydrogen in the most common fuel cells based on proton exchange membranes.

### 2.3. Hydrogen Production from Alcohols

From this perspective, liquid biomass products, including ethanol and methanol, are worth considering (Figure 1) [55,86,87]. The advantage of alcohols steam reform (proceeding according to Equations (6) and (7)) is characterized by significantly lower temperatures than the natural gas conversion process:

C_2_H_5_OH + 3H_2_O = 2CO_2_ + 6H_2_, ΔH° = 157 kJ/mol (6)

CH_3_OH + H_2_O = CO_2_ + 3H_2_, ΔH° = 50 kJ/mol (7)

These hydrogen production methods can be considered as renewable because the amount of carbon dioxide released is equivalent to that of the amount absorbed by the growing plants [81,88]. In the catalytic mode, the ethanol steam reforming runs at 400–500 °C, and the methanol conversion runs at 300–400 °C [89,90,91,92]. This allows for tuning the process to maximize hydrogen and CO_2_ yields while minimizing the CO concentration, which affects the efficiency of proton exchange membranes in fuel cells. Hydrogen production via partial ethanol oxidation is also investigated [93,94]. The process will be discussed in detail in the section covering membrane reactor application, for which it seems the most practical. There are also biochemical methods for hydrogen production from biomass, such as, for example, photo- and dark fermentation [95,96,97]. However, the efficiency of these methods is currently low.

### 2.4. Catalysts for Hydrogen Production

All of the above processes are carried out using catalysts. At the same time, despite the difference in chemicals and process temperatures, most of the catalysts (with the exception of methane pyrolysis) share a similar nature, since the C–H bond activation is a mandatory condition for these processes. Precious metals (Ru-Pd and Os-Pt) [64,98,99,100] have the highest activity in these processes. However, cheaper transition metals (Ni, Cu, etc.) are frequently used for these purposes [101,102,103,104,105,106,107,108,109,110]. Remarkably, for processes such as the steam reforming of alcohols, which occur at relatively low temperatures, the choice of transition metals (nickel and copper) as catalysts can provide higher CO_2_ selectivity and suppress the formation of CO and coke on the catalyst surface [31,111]. Bimetallic catalysts are even more productive [112,113,114,115].

**Figure 2:**
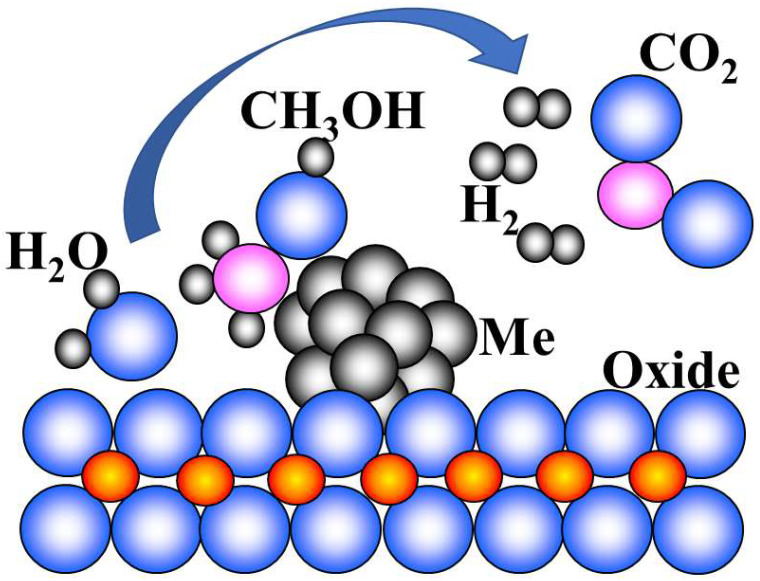
Scheme of catalytic conversion in the process of methanol steam reforming on a metal catalyst with an oxide carrier.

To improve the performance of the catalysts, they are deposited on supports, primarily aluminum, silicon, zirconium oxides, or more complex oxide systems (Figure 2) [99,116,117,118,119]. Carbon supports are used less often [120,121]; in some cases, they can promote more efficient steam reforming processes, minimizing carbon monoxide generation [122,123]. For a number of systems, the supports perform their own function, such as water sorption in the alcohols or methane steam reforming processes (Figure 2) [124]. For carbon carriers, this can be achieved by using carboxyl and hydroxyl groups formed on their surfaces [122].

### 2.5. Water Electrolysis

In terms of the environment, the most promising hydrogen production method is water electrolysis, which not only allows for producing absolutely clean hydrogen in a single stage but does not lead to environmental pollution (Figure 1). However, the process is extremely energy-consuming. Even at low current, when the efficiency of the electrowinning cell is the highest, it would normally require at least twice as much energy as the amount of energy that can be generated from hydrogen produced in high-efficiency fuel cells. It is caused by the polarization loss in these devices [125,126,127,128,129]. Therefore, hydrogen produced by electrolysis is usually 2–4 times more expensive than hydrogen generated by natural gas reforming [20,130]. Moreover, with the power generated using standard processes, the integral emissions of carbon oxides are very high. Therefore, the only promising option is to produce hydrogen from the renewable energy sources [131].

There are three main types of electrolyzers, which are alkaline, proton exchange membranes, and solid oxide electrolyzers [132,133,134,135,136,137,138,139,140]. Microbial electrolyzers are also developing, which operate at a significantly lower potential difference (0.2–0.8 V) due to organic substances being oxidized by electrogenic bacteria at the anode, and not oxygen release. However, the process cannot be commercialized due to its low efficiency [141,142].

The main factors determining the advantages and disadvantages of various hydrogen production methods are summarized in Table 1. Based on an environmental impact, including carbon oxide footprint by various hydrogen production processes, color coding has been adopted in the scientific publications to characterize the colorless hydrogen [143,144]. It is this factor that considerably defines the prospects for various hydrogen production methods. Thus, despite the coal gasification being the cheapest technology, the high level of impurities narrowed its application [50,52,145,146,147,148,149,150]. On the contrary, more expensive electrolyzers powered by renewable energy sources are considered the most promising [20,50,133,151]. At the same time, there is an opinion that, as early as by 2030, such sustainable “green” hydrogen may become cheaper to produce vs. hydrogen from coal and gas [127].

## 3. Palladium Membranes for Hydrogen Purification

### 3.1. Hydrogen Purification Methods

High-purity hydrogen is traditionally in demand in the microelectronics and chemical industries. Thus, the demand for ultrapure hydrogen is expected to grow significantly, as low-temperature fuel cells, which dominate the current market, require hydrogen of exceptional purity, free from even trace amounts of carbon monoxide (CO). This is due to the fact that CO impurities drastically reduce fuel cell performance, since they are strongly sorbed on the surface of noble metal-based catalysts, causing irreversible deactivation of their active sites. Therefore, the hydrogen produced by most common processes, excluding water electrolysis, requires further deep purification. Currently, only 0.1% of globally produced hydrogen meets the purity standards required for these applications without additional purification [152]. The common processes for hydrogen purification are pressure swing adsorption (PSA), cryogenic distillation, and membrane separation [54,153,154,155,156]. PSA is the most widely used hydrogen purification technology, accounting for 85% of global hydrogen purification. The process involves passing hydrogen through adsorbers, which are capable of capturing impurities selectively (e.g., H_2_O, CO, CO_2_, and N_2_). Through cyclic adsorption and desorption driven by pressure changes, PSA can reduce impurity levels to below 0.05% [153,157,158,159].

Due to the extremely low hydrogen boiling point (about 20 K), cryogenic distillation can be effectively applied for hydrogen separating from other gases, especially in the case of liquid hydrogen storage. The impurities are removed during the initial liquefaction stage at moderately low temperatures. However, the high energy intensity of the process makes it expensive [160]. Moreover, cryogenic distillation is ineffective for reducing the impurity level below 5%, making it unsuitable for producing hydrogen purities required for low-temperature fuel cells [48,156].

Interestingly, the hydrogen can also be purified using hydrogen storage techniques, such as metal hydride technology, and liquid carriers [76]. At the same time, their wide application is limited by a number of disadvantages. For instance, the alloys used for hydrogen storage are subjected to catalytic poisoning, similarly to the catalysts in fuel cells. As a result, these techniques are best suited for hydrogen storage and transportation rather than direct purification for end-use applications.

Membrane separation is considered one of the advanced hydrogen purification methods [161,162,163]. Hydrogen separation membranes can be classified into four main types: organic (polymeric), inorganic, hybrid, and electrochemical membranes [156,164,165,166,167]. Polymeric membranes are the most commonly used for gas separation due to their low cost, high performance, and scalability. The separation performance in polymeric membranes is governed by the dissolution–diffusion mechanism [162,168,169]. However, the high separation on the molecular level cannot be achieved in the polymeric membranes due to the disorderly nature of polymeric materials. To enhance the selectivity and permeability of polymeric membranes, hybrid membranes incorporating zeolites or metal–organic frameworks (MOFs) have been developed. These materials improve hydrogen permeability and separation performance in comparison to conventional polymer membranes [170,171]. Recently, MOFs have been considered as promising materials for hydrogen purification due to the presence of porosity of a strictly defined size. In this case, the molecular sieve effect is used, similarly to carbon materials. A wide choice of metal ions and organic ligands provides the desired properties, size, and structure of the pores [172,173]. The authors of [174] proposed an original concept for the limited growth of MOFs inside a supported layer of covalent organic frameworks (COFs) for the preparation of membranes. The combination of high hydrogen permeability together with a significant increase in the selectivity of its separation with gases such as methane and carbon dioxide determines their excess of the upper bound in the Robson plot. The membranes with carbon-based structures, such as graphene, carbon nanotubes, and molecular sieves, provide attractive separation on a molecular level. Meanwhile, producing defect-free carbon membranes with uniform pore sizes remains technically challenging. From this point of view, MOFs with well-defined pore sizes seem more attractive, but these pores are usually large enough compared to small molecules to ensure complete hydrogen separation from gases such as helium and some others. In addition, it is also difficult to fabricate large, defect-free membranes in one piece. Despite these limitations, carbon and MOF-based membranes are increasingly used for separating hydrogen from other gases, such as nitrogen, methane, and carbon dioxide [153,175,176,177]. Compared to these materials, metal membranes provide low defectiveness. In addition, hydrogen transfer in them occurs through dissolution and diffusion. This mechanism is unattainable for other gases, which determines extremely high selectivity. At the same time, the comparatively low rate of hydrogen transfer in palladium membranes forces one to look for alternative solutions to accelerate hydrogen transfer.

### 3.2. Palladium Alloy-Based Membranes

Highly selective hydrogen separation can only be achieved using metal membranes. The certain metals enable the dissociative sorption of hydrogen, which can be diffused into the metal matrix in atomic or even ionic form as a result of the integration of hydrogen electrons into the electronic system of the metal [178]. Among these, palladium membranes are the most widely studied and used. However, other metals, such as vanadium, niobium, tantalum, and tungsten, exhibit even higher hydrogen permeability [11,179]. Unfortunately, these metals are prone to rapid oxidation, forming oxide layers that, even having extremely small thickness, block hydrogen transport. Additionally, these metals possess a tendency of hydrogen embrittlement, which limits their applications for hydrogen purification processes. As a result, despite relatively lower hydrogen permeability and high cost, palladium is typically preferred for the production of highly pure hydrogen due to its superior stability [11,58,180,181,182,183,184,185].

Nevertheless, palladium is not without its challenges; for example, at 295 °C, palladium undergoes a phase transition between α- and β-phases, accompanied by a 10% change in the volume of its face-centered cubic (fcc) lattice. This can compromise membrane integrity during thermal cycling [186]. This phase transition can be mitigated by reducing the particle size or by alloying palladium with other elements [187]. Moreover, the palladium membranes can be poisoned by impurities such as carbon monoxide, hydrogen sulfide, and unsaturated hydrocarbons, which block active sites on the membrane surface, obstructing the adsorption of hydrogen [188,189,190]. Furthermore, relatively low hydrogen permeability of palladium, which is dominated by volume diffusion, requires the use of thin films. These thin membranes often lack sufficient mechanical strength and may fail under pressure differentials.

To overcome these limitations, several strategies have been developed to improve the performance of palladium membranes, including alloy formation, composite material development, and surface modification [191]. One of the most common approaches to improving palladium membranes is alloying with metals such as silver, copper, gold, ruthenium, and others. The main goal is to suppress phase transitions, enhance hydrogen permeability, and partially reduce costs by replacing some of the expensive palladium with alternative metals.

Palladium–silver alloys, typically containing 20–25% silver, are widely used. These alloys shift the phase transition temperature closer to room temperature, significantly increase hydrogen permeability, and improve resistance to hydrogen embrittlement. However, membranes based on these alloys are more susceptible to poisoning by carbon monoxide (CO) and hydrogen sulfide [11,178,189,192,193,194,195]. The alloys doped with more expensive gold have similar properties [196,197,198,199,200]. Copper-doped palladium membranes are another common approach. Despite the electronic configuration similarity between copper and silver, their influence on membrane properties differs significantly. The copper doping improves hydrogen permeability and stability, including at high temperatures, and makes membranes less sensitive to poisoning by CO and unsaturated hydrocarbons. At the same time, hydrogen sulfide damages these membranes within a shorter time [184,197,201,202,203,204,205,206]. In our opinion, this observation suggests that the reduced stability of silver- and gold-doped membranes to CO poisoning may not be due to a reduction in active sites, as some researchers suggest, but rather may be due to changes in the Fermi level and the chemical affinity of the membrane surface for toxic agents.

Ruthenium alloys are also in demand, which have a distinctive feature: the higher hydrogen permeability at low temperatures (to 200 °C) and the greater resistance to H_2_S. However, these alloys exhibit lower selectivity at higher temperatures, probably due to loss of membrane integrity [207,208,209,210,211,212]. The yttrium-doped palladium membranes display the highest hydrogen permeability; meanwhile, these materials corrode rapidly in the presence of oxidizing agents and are susceptible to CO poisoning [202,213,214].

The electronic configuration of palladium is similar to that of nickel, and a few publications have reported the application of nickel membranes in the deep purification of hydrogen at fairly high temperatures of 600–900 °C [215]. Consequently, palladium–nickel alloys have also been tested and reported in the literature for hydrogen purification [216,217,218]. Unfortunately, these alloys exhibit lower hydrogen permeability and a higher tendency to be poisoned by CO and CO_2_ [217].

The study of ternary palladium alloys is an active area of research. For instance, palladium doped with copper subgroup metals (e.g., Cu-Ag-Au) or nickel doped with gold can enhance membrane stability and the hydrogen permeability [184,219]; however, this approach cannot overcome the problem of the material stability toward hydrogen sulfide [220]. Significant improvements in hydrogen permeability can be achieved with Pd-Ru-In [221] and Pd-Ag-Y [222] alloys at 350 °C, even with low concentrations of indium and yttrium, respectively.

### 3.3. Surface Modification of Palladium Membrane

Palladium-based membranes are typically fabricated using cold rolling techniques [206,223]. However, residual lubricating oils from the rolling process, as well as the adsorption of nitrogen, carbon oxides, microdroplets of liquid from the air, and dust, can partially block the membrane surface. Since the sorption and desorption of hydrogen on the membrane surface are critical steps in the diffusion process [224], these impurities can significantly reduce hydrogen permeability by blocking the active sites, similar to the poisoning of fuel cell catalysts [225]. Furthermore, these impurities are often difficult to be removed using conventional cleaning methods, such as washing with acetone or alcohols, due to the high sorption activity of precious metals toward certain molecules, including those with multiple bonds or sulfur groups. In some cases, the solvents can also be strongly adsorbed onto the membrane surface. To address this, heat treatment is commonly employed to clean the membrane surface and optimize its phase composition prior to use [226,227,228]. Additionally, advanced surface activation methods, such as photon or ultrasonic processing, have been shown to be even more effective in removing impurities [229,230,231].

**Figure 3:**
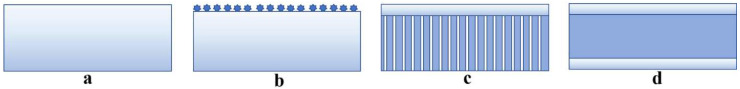
Scheme of membrane structures for hydrogen purification: palladium alloy membrane (**a**), nanostructured coated membranes (**b**), membrane with a thin selective layer (**c**), and hydrogen permeable metal composite membranes with a palladium coating (**d**).

Another promising approach involves increasing the membrane surface area, which is commonly used for polymeric membranes [232]. This method may first appear impractical for palladium containing membrane applications, where hydrogen transfer is usually limited by diffusion. Nevertheless, high-performance plants require the palladium layer thickness to be reduced. This increases the diffusion rate, while proton transfer is limited by the hydrogen sorption and desorption processes [233]. An increase in the surface area accelerates these processes. One effective method involves coating one or both sides of the membrane with a highly porous nanostructured layer of the same composition (Figure 3b) [234,235]. This technique has parallels in the fabrication of standard hydrogen electrodes and has been shown to enhance palladium catalytic activity in some electrochemical processes, such as the electrooxidation of alcohols. The electrolytic oxidation of alcohols, which has found its application in direct methanol fuel cells [236,237,238], is a good example.

The crystal surface of metals is inherently heterogeneous, with different faces exhibiting varying sorption and catalytic activities. The most active surfaces are those with a lower packing density and higher energy levels [239,240]. Consequently, increasing the number of such high-energy faces on the surface of palladium-based membranes enhances the hydrogen purification rate [187]. A particularly interesting example involves membranes coated with star-shaped palladium nanoparticles [241,242,243,244]. Obviously, symmetry involving fifth-order axes cannot be realized in crystal structures, but it could be developed because of multiple twinning [245], resulting in higher activity of particle surfaces.

For instance, palladium nanostars developed on a 10 µm Pd-23%Ag film surface achieved a record increase in hydrogen permeability, reaching up to 12.5 µmol s⁻^1^ m⁻^2^ at just 100 °C [246]. After coating, the activation energy for hydrogen permeability dropped from 72 to 51 kJ mol⁻^1^, both of which were significantly higher than the values cited in publications focused on diffusion-limited membranes [247]. This indicates that hydrogen transport in these membranes was limited by sorption processes, which were significantly enhanced by surface modification. Notably, the coatings also exhibited high peak current density values of up to 238 mA cm⁻^2^ in the methanol alkaline oxidation reaction, which was four times higher than the activity of standard palladium nanoparticle coatings, despite both methods producing particles of similar size [246].

### 3.4. Composite Membranes

The previous section has shown that chasing an endless decrease in the thicknesses of palladium membranes is pointless, since transfer in very thin membranes is limited by sorption activity. In addition, a higher pressure drop between the permeate and retentate would be required to increase the hydrogen filtration rate. Unfortunately, thin films cannot withstand such a pressure drop. To deal with this obvious dilemma, composite membranes were introduced a long time ago with a thin selective palladium alloy layer applied onto a porous substrate (Figure 3c), which enhances the membrane strength without limiting transfer processes [248,249,250].

Again, the composite membranes have their own limitations, such as that they are problematic to manufacture, as such a thin layer cannot be applied onto a substrate with a large pore size (i.e., high roughness). This becomes even more critical for metallic porous substrates, for which achieving a small pore size is an issue. The mentioned problems, however, are much easier to deal with in ceramic carriers, among which aluminum oxide is the most popular [181,251,252,253,254,255,256].

Such membranes display hydrogen permeability in the range of 3.6–6.3 mol·m^−1^·s^−1^·Pa^−0.5^, or higher in the case of Pd-containing alloys (Table 2). Herewith, H_2_/N_2_ separation selectivity may exceed 10,000. For example, for a 5 μm palladium layer coated on Al_2_O_3_, hydrogen permeability at 350 °C of 4.9 × 10^−9^ mol·m^−1^·s^−1^·Pa^−0.5^ and selectivity for the H_2_/N_2_ pair of 8000–37,600 were achieved [257]. Yttrium-stabilized zirconium oxide was used as a substrate for the Pd-Au alloy membrane [258]. Interestingly, the authors could not detect any degradation of the membrane in the presence of CO. However, palladium membranes with a porous ceramic substrate have their own problems, such as different coefficients of thermal expansion (CTE) and low contact strength due to a low adhesion capacity.

Hence, despite the existing issues, the most popular are stainless steel substrates as they are less CTE-sensitive [251,259,260]. For example, a 10 μm Pd-Ag layer applied onto porous stainless steel (PSS) base at 450 °C displayed hydrogen permeability of 3.4 × 10^−8^ mol·m^−1^·s^−1^·Pa^−0.5^ and H_2_/N_2_ pair selectivity of 39,000 [194].

Cases of application of composite membranes with palladium coating applied onto porous polymer substrates have been discussed. Their application for hydrogen purification is described by the authors of [261]. As temperature conditions for such membranes must be limited, they are frequently used for other purposes, such as hydrogenation processes [262]. The application of such membranes for the removal of dissolved oxygen and organochlorine compounds from water will be discussed in the last section of this review.

To overcome the issues associated with applying a thin palladium layer on metal substrates due to problems with achieving a small pore size, ceramic interlayers are investigated as a solution to reduce the surface porosity and roughness. The most promising approach is applying intermediate layers onto cerium oxide with a coefficient of thermal expansion close to both palladium and stainless steel [263]. The highest performance in terms of hydrogen permeance (6.0 × 10^−4^ mol·m^−2^·s^−1^·Pa^−0.5^) was achieved by using an intermediate layer composed of CeO_2_ particles with intermediate sizes (34 μm on average), which allowed for formation of a dense palladium layer with thickness of 6.3 μm, although the highest permeability was observed for the membrane containing CeO_2_ particles with smaller sizes (Table 2). High hydrogen selectivity was also reported for these membranes [264]. Martinez-Diaz et al. [265,266] discussed the advantages of using mesoporous CeO_2_ to make membranes with high permeability and hydrogen selectivity. Graphite [267], graphene oxide [268], and various zeolites [269,270,271] were also employed as intermediate layers.

As noted above, some metals have significantly higher permeability compared to palladium but cannot be used due to high degradation in the oxidizing atmosphere. They can also be used as a kind of non-porous carrier. Actually, such metals can form a membrane themselves, and palladium, in this case, will rather act as a protective coating, which can be extremely thin (Figure 3d) [272]. Hydrogen permeability of the membrane studied in the work [273] was (6–12) × 10^−8^ mol·m^−1^·s^−1^Pa^−0.5^ at 450 °C. The palladium layer thickness was only 1 μm, and the membrane hydrogen selectivity was absolute. Surprisingly, tantalum membranes with a Pd-Cu coating displayed lower hydrogen permeability and higher stability compared to a pure palladium coating [274]. Tubular membranes based on V-Pd and Fe-Pd alloys with palladium coating were studied in [275,276,277,278]. A disadvantage of the membranes based on metal pairs is mutual diffusion, resulting in the membrane surface degrading due to the oxidation of more active metal appearing on the surface. This is why the systems also use intermediate layers to prevent diffusion. For example, graphene [279] and hafnium nitride [280] were used to manufacture such layers.

**Table 2:** Examples of high-temperature f composite membranes based on palladium layer supported on porous substrates.

Membrane	*T*, °C	Pd Layer Thickness, μm	H_2_ Permeability, 10^−9^ mol·m^−1^·s^−1^·Pa^−0.5^	H_2_/N_2_ Separation Selectivity	Ref.
Pd/Al_2_O_3_	350	5	5.0	8000–37,600	[257]
Pd/Al_2_O_3_	400	5	6.3	–	[257]
Pd-Ag/PSS	450	10	34	39,000	[194]
Pd/PSS-CeO_2_ (0.07–0.1 μm)	400	12.5	4.5	>10,000	[264]
Pd/PSS-CeO_2_ (3.4 μm)	400	6.3	3.8	>10,000	[264]
Pd/PSS-CeO_2_ (>10 μm)	400	9.0	3.6	>10,000	[264]
Pd/Ta/Pd (tubular)	450	1	60–120	–	[273]

Applications of polymer materials are constrained by the low thermal stability of most polymers. However, there are a few publications reporting palladium membranes on a polymer substrate. The first example of such membranes are [281,282,283], in which composite membranes based on polyethylene terephthalate (PET), polytetrafluoroethylene (PTFE), and cellulose acetate (CA) coated with a thin palladium layer were studied, as well as the works of V.M. Gryaznov, in which the hydrogen permeability through a number of polymer membranes coated with palladium alloys was discussed [284,285]. The palladium layer coating on a PET and PTFE membrane significantly reduces permeability for all gases, but the hydrogen permeability can remain at a sufficiently high level. As a result, with a palladium layer coated with a polymer membrane, the separation selectivity of hydrogen-containing gas pairs may go 10 times higher. H_2_/N_2_ separation selectivity above 150 and H_2_/CO_2_ separation selectivity above 10 are achieved for CA/natural rubber membranes with a continuous palladium layer. The advantage of polydiphenylene phthalide-based membranes with a palladium-based alloy layer containing 6% ruthenium is higher hydrogen permeability at high temperatures compared to a pure polymer membrane, despite the fact that the pure polymer membranes had higher permeability at room temperature [285].

Several examples of polymer membranes with palladium plating based on polycarbonate [286] and polybenzimidazole with hexafluoro-propylidene diphenyl fragments in the backbone (PBI-HFA) are described [261]. At 150 °C and a gas pressure of 8 bar, the H_2_/CO_2_ selectivity for Pd-coated PBI-HFA membrane is up to 5 times higher than selectivity of a pure polymer membrane, with the hydrogen permeability slightly reduced (Table 3). Metal layer gas transfer properties and morphology for these membranes strongly depend on how palladium was coated on the polymer layer, the substrate selected, and the polymer processing method used before applying the metal layer. Vacuum coating provides a solid defect-free Pd-layer. An important feature shared by these membranes is that they are all completely impermeable to carbon monoxide. This provides an opportunity for such membranes not only to be used for hydrogen purification but for syngas component separation as well.

One of the processes used to create high-performance membranes for hydrogen-containing mixture separation is a palladium coating of track membrane pore surfaces [287]. Typically, track membranes are not used for gas separation because they have through-pores with a diameter much larger than the gas molecule diameter, and, therefore, have very high gas permeability, but not selective. However, the modification of such palladium membranes can help to increase H_2_/CO_2_ separation selectivity [288,289].

Palladium nanoparticles have also been introduced directly into the polymer matrix [290]. Such membranes can have better performance and lower cost compared to membranes with continuous palladium layer and a higher selectivity for hydrogen-containing gas pair separation in contrast with polymeric membranes [291,292].

When selecting a polymer material for such membranes, its thermal stability and gas transport characteristics play an important role [293]. It is advisable to fabricate the hydrogen containing gas mixture separation membranes based on the polymers characterized, on the one hand, by moderate or high gas permeability, and, on the other hand, by high glass transition, decomposition, and melting temperatures. Therefore, the range of heat-resistant polymers used to create gas separation membranes should be discussed separately. Thermally stable polymers are normally characterized by a high molecular weight, rigid and inactive backbones, the presence of structural fragments resistant to high temperature chemical processes, and structural fragments that provide a sufficiently strong intermolecular interaction (hydrogen bonds and dipole–dipole interactions) [294]. These include fluorine-containing polymers [162,295,296]; inorganic backbone polymers (polysiloxanes and polyphosphazenes) [297,298,299,300,301]; rigid backbone carbocyclic polymers, such as polyamides, polysulfones, and polynorbornenes [302,303,304]; and related heterocyclic polymers, e.g., polyimides and polybenzimidazoles [305,306,307,308,309,310]; as well as “ladder” polymers with rigid backbones and conjugate bonds in the backbone (e.g., polypyrrone) [293]. Some examples of heat-resistant polymers used for membrane development and their main characteristics are presented in Table 4. Among glassy polymers, heterocyclic polymers (polyimides, polybenzimidazoles, polybenzoxazoles, polyoxadiazoles, etc.) demonstrate the best thermal stability [311,312,313,314,315]; however, they are generally characterized by relatively low gas permeability due to a low free volume caused by dipole–dipole interactions [316]. Fluorinated polymers exhibit both high gas permeability and high thermal stability [295]. However, their disadvantage is the high cost. The thermal stability of carbocyclic polymers can be increased by introducing heterocyclic structural fragments into the side chain, like for polynorbornenes [317].

There are also some new polymer classes being developed, including thermally-rearranged polymers [323,324], polymers of intrinsic microporosity [325,326,327,328,329], vinyl-addition polynorbornenes [317,330,331,332,333,334,335,336,337], poly(hydrazide-imides) [319], and poly(naphthoylenbenzimidazoles) [316,338].

Polybenzimidazoles stand out among the above polymers, as they are inherently characterized by high H_2_/CO_2_ selectivity during high-temperature gas separation, resistance to plasticization, and stability at temperatures up to 425–435 °C [307]. There are publications describing examples of hybrid membranes with Pd nanoparticles based on *m*-polybenzimidazole (*m*-PBI), as well as polyimide and polyamidimide.

**Figure 4:**
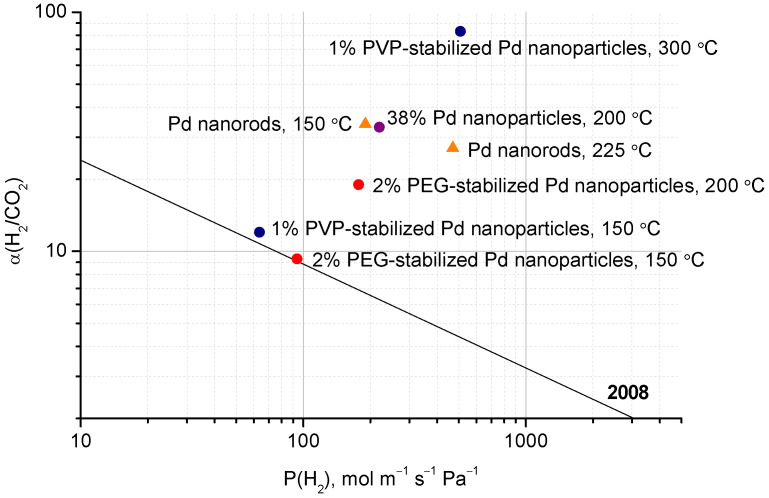
Robeson Plot for H_2_/CO_2_ pair and parameter positions of some composite membranes based on *m*-PBI with Pd nanoparticles [339,340].

The introduction of polyethylene glycol-stabilized Pd nanoparticles into a *m*-PBI polymer matrix somewhat decreases the membrane’s hydrogen permeability, which is likely due to metal particles impeding the mass transfer in the polymer micropores. However, the transfer of other non-soluble in palladium gases is blocked to a much greater extent. Therefore, the selectivity of H_2_/N_2_ and H_2_/CO_2_ separation is increased by up to 30 and 5 times, respectively [339]. This puts the composite membrane parameters above the upper bound on the Robeson plot for the H_2_/CO_2_ gases pair (Figure 4). For similar membranes based on *m*-PBI with Pd nanoparticles stabilized with polyvinylpyrrolidone (PVP), the effect is the same (Table 5) [340]. In [341,342], it is shown that hybrid membranes containing Pd nanoparticles demonstrate high H_2_/CO_2_ selectivity at high temperatures (up to 33 @ 150 °C).

At the same time, with Pd nanoparticles introduced into a *m*-PBI hollow fiber-based membrane, the selectivity of H_2_/CO_2_ separation and hydrogen permeability is increased [343] (Table 5). Similarly, the introduction of *m*-PBI palladium nanorods into the membrane increases the hydrogen permeability and improves the H_2_/CO_2_ selectivity separation, which exceeded 30 @ 150 °C (see Table 5) [344].

**Table 5:** Gas transport properties of PBI-based membranes loaded with palladium nanoparticles.

Membrane	*T* (°C)	*P*(H_2_), 10^−16^ mol m^−1^ s^−1^ Pa^−1^	*Q*(H_2_), GPU	Separation Selectivity		Ref.
				H_2_/N_2_	H_2_/CO_2_	
*m*-PBI	150	150	–	3.4	1.4	[339]
	200	260	–	5.0	4.1	[339]
*m*-PBI + PEG-stabilized Pd nanoparticles (2%)	150	94	–	68	9.3	[339]
	200	180	–	81	19	[339]
*m*-PBI + PEG-stabilized Pd nanoparticles (3%)	150	210	–	4.1	3.5	[339]
	200	320	–	7.3	5.8	[339]
*m*-PBI + PEG-stabilized Pd nanoparticles (4%)	150	94	–	75	6.4	[339]
	200	210	–	110	5.0	[339]
*m*-PBI	100	43.5	–	4.8	2.5	[340]
	150	57	–	2.9	8.1	[340]
*m*-PBI + PVP (1%)	100	15	–	5.6	1.1	[340]
	150	47	–	3.2	1.8	[340]
*m*-PBI + PVP-stabilized Pd nanoparticles (1%)	100	16	–	5.4	2.4	[340]
	150	63.5	–	22	12	[340]
	200	160	–	39	21	[340]
	240	160	–	43	21	[340]
	260	315	–	83	58	[340]
	300	510	–	180	83	[340]
*m*-PBI + PVP-stabilized Pd nanoparticles (3%)	100	6.0	–	5.9	2.9	[340]
	150	30.5	–	8.7	6.0	[340]
*m*-PBI + Pd nanorods	150	190	–	–	34	[344]
	225	470	–	–	27	[344]
*m*-PBI + Pd nanoparticles (58 wt%)	200	220	–	–	33	[341]
*m*-PBI (in hollow fibers)	60	–	0.086	–	5.2	[343]
*m*-PBI + Pd nanoparticles (in hollow fibers)	60	–	80	–	10	[343]

In [345,346], a series of polysulfone-based membranes containing varying amounts of palladium nanoparticles was developed (Table 6). Compared to a pure polysulfone-based membrane, such membranes demonstrated one order higher hydrogen permeability and, at the same time, a higher selectivity of H_2_/N_2_ separation (15–20 for Pd-containing membranes vs. 6.2 for pure polymer membrane). The largest increase in H_2_-containing gas pair separation selectivity was observed at a Pd content of 2 wt% [345]. Another example is polycarbonate composite membranes with aligned carbon nanotubes modified with palladium nanoparticles [347]. Compared to similar palladium-free membranes, such membranes exhibited lower permeability, with higher selectivity of H_2_/CO_2_ separation observed when using oxidized nanotubes. Composite membranes based on Matrimid polyimide modified with ZIF-8 and Pd nanoparticles were also characterized by higher hydrogen permeability and close permeability to other gases compared to similar membranes without Pd nanoparticles, resulting in 1.5 times higher selectivity of H_2_/N_2_ and H_2_/CO_2_ separation [348].

Gas separation composite membranes based on natural polysaccharide (guar gum) with graphene oxide [349] and chitosan [350] additives, modified with palladium nanoparticles, have been developed. With palladium nanoparticle introduction, the opposite effect was observed—a decrease in gas permeability, and the most drastic one was for hydrogen. As a result, selectivity inversion occurs, and the modified membranes are characterized by a fairly high selectivity of CO_2_/H_2_ separation (24 with graphene oxide and 13 with chitosan) with a CO_2_ permeability of 1.1 × 10^−11^ and 4.0 × 10^−12^ mol m^−1^ s^−1^ Pa^−1^, respectively. There is also an example of modifying a membrane based on cellulose acetate with palladium acetate [351]. Unlike the modification with palladium nanoparticles, such modification leads to a membrane permeability increase for H_2_ and CO_2_, while the selectivity of CO_2_/CH_4_ and H_2_/CH_4_ separation increases by 1.6–1.9 times.

## 4. Membrane Reactors

The concept of membrane reactors involves integrating chemical synthesis with the deep purification of a target product by separating it from the reaction system through a membrane. For hydrogen production, this approach can significantly simplify the process flow (Figure 1). The membrane reactors can also be used for the selective catalytic conversion of precursors into target products by uniformly supplying one of the reactants. Currently, a wide range of membrane reactors has been developed applying various types of membranes, including polymeric, porous, and non-porous oxides, zeolites, and others. There are also enzymatic, biomedical, photocatalytic membrane reactors. In addition, devices based on ion-exchange membranes can be classified as membrane reactors. Although these devices often perform different functions, they are widely used in the energy and chemical industries—for example, in fuel cells, electrolysis cells, electrodialysis, and reverse electrodialysis systems.

**Figure 5:**
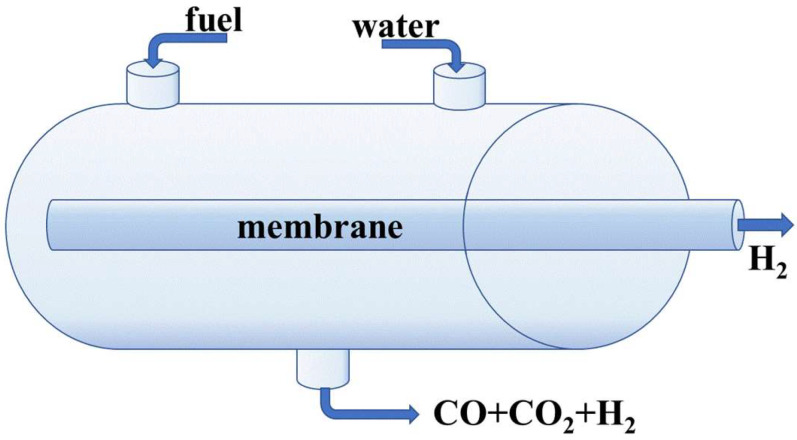
Scheme of hydrogen production using membrane reactor.

The concept of membrane reactors was first proposed by Gryaznov and co-workers [13]. Since then, numerous designs have been developed, featuring various membrane geometries, such as flat, radial, and tubular membranes in linear or coiled configurations. However, membrane reactors are more commonly classified based on the membrane function. In the extractor reactors, the membrane selectively removes products from the reaction mixture, shifting the thermodynamic equilibrium toward higher yields. In the membrane extractor, the membrane controls the feed of reactants into the reaction mixture. This is particularly important for processes such as the partial oxidation of natural gas, where a uniform feed distribution prevents explosions and improves process selectivity. In the membrane contactor, the membrane enhances the contact between substrate and the catalyst, increasing the conversion degree [352]. Due to their unique properties, palladium-containing membranes are most commonly employed for hydrogenation and dehydrogenation processes [180,353]. These processes are particularly relevant for hydrogen energy applications, such as hydrogen production from natural gas, alcohol, and a number of other reagents (Figure 5) [354,355,356,357].

Researchers generally focus on maximizing the benefits of membranes, such as reduced thickness, increased surface area, and optimized contact time [58,358]. This has led to significant interest in the membrane micro-reactors, which help to optimize mass transfer, thereby reducing concentration polarization, ensuring a high membrane surface-to-reactor volume ratio, and increasing target product reform and selectivity [128,359,360]. In particular, they have been used in processes for hydrogen production, such as methane and alcohol steam reform, cyclohexane dehydrogenation, etc. [361]. However, the scaling-up of these systems for industrial applications remains a challenge.

The attractiveness of membrane reactors lies in their ability to combine chemical synthesis with product separation and purification [353,362], or a combination of two chemical processes (8 and 9), in one of which the substance transported through the membrane (C) is a product, and, in the other one, it is a reagent.

A ⇔ B + C(8)

C + D ⇔ E(9)

Selective extraction of the product from reaction (8) shifts the equilibrium, significantly increasing product yield beyond that determined by the thermodynamic equilibrium. For example, if a dehydrogenation process is carried out with hydrogen transfer through the membrane, then a strong oxidizing agent as the reagent D in reaction (9) can help to carry out dehydrogenation with high yield. This can also significantly reduce the process temperature [122,124,230,352,363,364].

The membranes also enable the precise control over reactant concentrations in the reaction zone. For instance, the slow diffusion of hydrogen through the membrane provides a controlled and uniform supply, which makes oxidation processes occurring at high speed and heat release safe and significantly increases the selectivity of reactions 8 and 9. Diffusion through the membrane also allows reactant B and D concentrations to be independently controlled in the process (9), and their competitive adsorption on the catalyst is inhibited, which is often a major problem in a conventional reactor [365]. The concentration of reactant C can be controlled by initial reduction with the contact, starting to increase selectivity and raising it in the end to provide a high degree of conversion, allowing counter-current mode of reactants A, B, and D [353]. Finally, since palladium-containing membranes have high thermal conductivity, process energy coupling can occur, which is especially important if one process is exothermic and another is endothermic, or proceeds with low heat generation, but requires elevated temperatures.

### 4.1. Hydrogen Production Using Membrane Reactors

One of the most widely studied applications of membrane reactors is natural gas steam reforming, which has been implemented using composite membranes with selective palladium layers. Methane reforming efficiencies of over 80% have been reported in membrane reactors [58,198,354,356,357,366,367]. However, the direct application of membrane catalysis in this process is challenging due to the high operating temperatures, which makes palladium-containing membranes unstable [368,369].

To optimize gas utilization and minimize harmful CO emissions, which are detrimental to both human health and fuel cell catalysts, a water–gas shift (WGS) reaction is typically performed as a second stage at 200–450 °C. In this process, CO reacts with excess water vapor to produce additional hydrogen, which can then be extracted using a membrane reactor [370,371,372,373,374,375]. For example, the researchers in [212] explored methane steam reforming in a Pd-Ru membrane reactor with a counter-current configuration. It was shown that the hydrogen transfer rate increases with increasing membrane length, but the average hydrogen flux decreases, and a significant increase in methane conversion due to an increase in the permeate purge rate was ineffective. A commercial implementation of this process for producing high-purity hydrogen from methane was realized by Tokyo Gas [361]. At the same time, some authors prefer to release hydrogen, for example, through dense palladium-plated vanadium or tantalum containing membranes after cooling the mixture to an acceptable temperature [277].

The steam reforming of alcohols is another attractive application for membrane reactors. Alcohols can be obtained from biomass, offering a renewable and easily transportable energy source, with hydrogen yields of approximately 9% by weight. The steam reforming of alcohols occurs at relatively low temperatures, requiring less energy and producing CO_2_ as the main byproduct. It is also worth noting that the methanol reforming with subsequent hydrogen oxidation in fuel cells provides extremely high vehicle mileage on one refueling, and a rapid refueling otherwise [48]. The membrane reactor steam reforming significantly increases the alcohols conversion above the thermodynamically equilibrium and produces high-purity hydrogen in a single process stage [20,376,377,378]. Furthermore, due to the exponential increase in the chemical process rate and the palladium-containing membrane permeability vs. temperature, both the reforming degree and the hydrogen recovery ratio increase rapidly with a rise in temperature [228,379].

For example, the membrane reactors with palladium–silver, palladium–ruthenium alloys, and anodic aluminum oxide membranes with a selective palladium layer can achieve methanol reforming of 85–100% at high purity and a hydrogen yield of 40–97% at 200–350 °C [111,124,355,356,380,381,382,383]. At the same time, low CO concentrations in products are usually noted, and almost without a catalyst and membrane carbonization.

On the other hand, the ethanol steam reforming is more challenging due to the difficulty of breaking C–C bonds, which requires higher temperatures (400–600 °C). This often leads to reduced selectivity, higher CO concentrations, and, in some cases, carbon deposition, which can poison both catalysts and membranes. As a result, ethanol reforming performance in membrane reactors varies widely, with conversion rates ranging from 40 to 100%, and the high-purity hydrogen yield does not exceed 93% and can drop to 10% due to concentration polarization [384]. Similar to methanol steam reforming, this process uses a wide range of catalysts, including noble metals, nickel, cobalt, and copper, and palladium–copper and palladium–silver alloys have been chosen as membranes [385,386,387,388], as well as a range of composite membranes [389,390,391,392]. The steam reforming of another biomass fermentation product, acetic acid, was also carried out in membrane reactors with palladium–silver membranes at 400–450 °C [393].

Membrane reactors are also actively employed for hydrogen production from liquid carriers, such as ammonia. In particular, the process of ammonia decomposition attracts much attention from researchers, which is an equilibrium process, and therefore, the product is always contaminated by the ammonia, which is a catalytic poison for fuel cells with proton exchange membranes. The use of membrane reactors in this process significantly increases reforming rates, reduces operating temperatures, and produces high-purity hydrogen in a single step [362,394,395].

### 4.2. Polymer-Based Composite Membranes with Catalytically Active Palladium Particles for Hydrogenation/Dehydrogenation Processes

The palladium potential for application as a catalyst for various reactions, for example, hydrogenation, dehydrogenation, dehydrohalogenation, and cross-coupling, provides additional opportunities for palladium based hybrid membrane application in membrane reactors [180,224]. The palladium-based membrane application in membrane reactors used for hydrogenation and dehydrogenation processes can combine the catalysis and separation processes in one material, which, in turn, simplifies the design and reduces the size of reactors and, thereby, improves their efficiency [180].

Several membrane reactors based on metal–polymer composite are described in the literature. Metal–polymer composites with palladium nanoparticles have been most often used for the various gaseous and liquid organic compound hydrogenation in mild conditions using hydrogen gas [396,397,398,399,400,401,402,403,404,405,406,407,408,409,410,411] (Table 7). In the majority of cases, such membranes are fabricated by the treatment of a polymer with palladium(II) salts, followed by reduction or heat treatment.

Other works [397,399,400,405,411] describe the developed catalytically active polymer membrane reactors with palladium nanoparticles based on a variety of commercially available flat membranes for propyne to propene and propene to propane hydrogenation. Reactors based on hollow-fiber membranes modified with palladium nanoparticles have also been described, in particular, for a propyne and propadiene-containing mixture for propene hydrogenation [407] with 90–99% selectivity. Such a reactor demonstrated a high (up to 99%) 1-butene selectivity during the 1,3-butadiene hydrogenation reaction [408]. A reactor based on a metathesis diblock copolymer containing norbornene units with an organopalladium substituent was applied for ethylene, propylene, and 1,3-butadiene hydrogenation [401,402].

There are examples of Pd polymer composite-based membrane reactors for liquid phase hydrogenation, including the hydrogenation of methylenecyclohexane [398], sunflower oil [396], octene-1 [410], 3-hexyn-1-ol to 3-hexen-1-ol [406], indene, 1-dodecene, 4-isopropenyl-1-methylcyclohexene, and diphenyl acetylene, as well as NO_2_-group containing aromatic compounds [409].

Another group of processes is related to electron-withdrawing substituents’ reduction in organic compounds. Polymer membrane reactors with palladium nanoparticles are widely used for the hydrogen reduction of *p*-chlorophenol [412] and chlorobenzene [413]. Liquid-phase micro-flow membrane reactors based on nitrogen-containing polymers poly(4-vinyl-pyridine) and the copolymer of 4-vinylpyridine with N-isopropylacrylamide, as well as a polymer with pyridinium fragments in the backbone (modified with palladium nanoparticles for the hydrodehalogenation of halogenated aromatic hydrocarbons, using sodium formate as a reducing agent), have also been proposed [414].

Reactors based on polymers with sulfo-groups containing palladium nanoparticles were used to reduce 4-nitrophenol to 4-aminophenol with sodium borohydride [415]. The authors of [416] used a membrane reactor based on polyamidimide hollow fibers with a selective layer of polydimethylsiloxane modified with Pd nanoparticles for the same purpose.

The examples of the application of polymer–palladium composite membrane-based reactors for cross-coupling catalysis, such as the Suzuki and Suzuki–Miyaura reactions [417], are reported. For instance, a polyethersulfone-based membrane modified with palladium nanoparticles can achieve 100% selectivity in the formation of 4-nitro-biphenyl in the cross-coupling reaction of 1-iodine-4-nitrobenzene with phenylboronic acid [418]. A flow-through catalytic membrane reactor based on a polyethersulfone composite containing iron nanoparticles with grafted catalytically active palladium nanoparticles demonstrated high efficiency in reactions between halogen-substituted arenes and olefins/acetylenes, as well as a reduction reaction of 4-nitrobenzene [419].

Thus, it can be concluded that polymer–palladium composites are promising materials for the membrane reactors used in a wide range of processes. We can expect the further development of high-performance, highly selective, and reliable membrane reactors based on nano-palladium polymers.

### 4.3. Polymer–Palladium Composites in Fuel Cells

**Figure 6:**
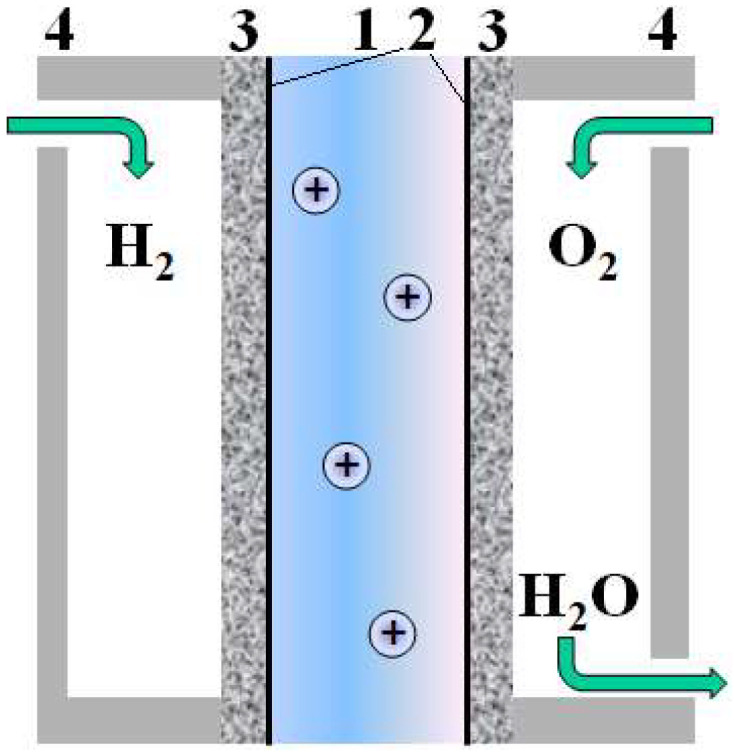
The scheme of hydrogen–oxygen FC with proton exchange membranes. Proton exchange membrane (1), catalyst (2), gaseous diffusion layer (3), and bipolar plates (4) (reproduced with permission from [48]).

One of the most important directions of polymer–palladium composites’ potential application is the development of materials for fuel cells and other electrochemical devices. In fact, a fuel cell (FC) is a membrane reactor, although it differs significantly from those previously discussed in terms of functions, since its end product is electric power. The application of monodispersed metallic catalysts over proton- or anion (oxygen or OH^−^) -conductive membranes (Figure 6) can achieve high fuel cell productivity and efficiency [420]. Vice versa, the electric conductivity and gas permeability (permeability to hydrogen, oxygen, or other hydrogen-containing fuels, such as methanol) of these membranes should be very low to prevent energy losses [48]. Furthermore, both the membrane and the catalyst should be extremely stable. This is why in the most common proton exchange fuel cells, Nafion perfluorinated membranes and their analogues with a shorter side chain (e.g., Aquivion) are mostly demanded [421,422,423]. Normally, such FCs use platinum catalysts that combine high oxygen electro-oxidation catalytic activity and hydrogen electro-reduction [424,425].

The high platinum cost and its poor resistibility to poisoning by carbon monoxide prompt the search for a partial or complete replacement with platinum catalysts. There is evidence in the literature that palladium-containing catalysts are subjected to carbon monoxide poisoning to a lesser extent [426]. Also, in some cases, palladium-based catalysts are more active than platinum ones in both hydrogen–air and direct methanol and ethanol fuel cells [22,427,428]. The palladium nanoparticles additive to the membrane often helps to significantly reduce the methanol crossover of Nafion membranes without significant losses in proton conduction and vice versa, and, in some cases, the latter is even reduced [420,429,430]. Methanol and gas permeability decrease is not a palladium feature, but it is rather typical for hybrid membranes. This feature is explained by the theory of limited elasticity of membrane pore walls [431], which states that the introduction of nanoparticles into the ion-exchange membrane pores increases the volume and size of their channels controlling their conductivity. At the same time, a part of the electroneutral solution, which plays a significant role in gas and methanol molecule transfer, is displaced from the pore centers. In this case, the palladium advantage is that it simultaneously acts as a catalyst for the electro-oxidation of alcohols.

A comparison in methanol conductivity and permeability values changes for such membranes is shown in Table 8. Perhaps the best results were achieved for the palladium-impregnated Nafion 117 membrane prepared with the treatment of the polymer with the Pd(II) salt solution in supercritical CO_2_ with the subsequent reduction of Pd(II) to Pd(0). The methanol permeability in such membranes decreased by 7.5 times [432]. In [433], the synthesis of sulfonated polyether ether ketone (SPEEK)-based membranes, modified with graphite oxide with palladium, characterized by increased ionic conductivity and reduced methanol permeability, was described. These membranes are less popular due to the lower stability and ionic conductivity of SPEEK compared to Nafion.

The authors of a number of works have shown high activity of palladium and Pt-Pd containing catalysts plated on polyvinyl carbazole, polypyrrole, polyaniline, and poly(1,8-diaminonaphthalene). In some cases, oxides of vanadium, tin, tungsten, manganese oxyhydroxide, graphene, or graphene oxide particles are simultaneously introduced into a membrane [454,455,456,457,458,459,460]. In this case, materials that differ in their own conductivity should be preferred. The use of oxide carriers (gas diffusion layers) in fuel cells in some cases helps to increase the catalysts’ stability, preventing the triple contact destruction where electrocatalytic transformations usually take place [461]. For example, the Pt-Pd nanoparticles’ oxide and polyaniline-based catalyst for the electro-oxidation of methanol lost no more than 10% of activity in 70 h [457].

There are examples of palladium-based fuel cell catalysts with other metals, such as the Pd-Co composite with N-doped reduced graphene oxide and polyaniline [462], which are characterized by high catalytic activity in methanol oxidation. Flexible electrodes for ethanol fuel cells are based on palladium deposited on silver nanowires with polydimethylsiloxane [463].

In addition to fuel cells, polymer–palladium composites can be promising for devices related to other electrochemical processes, such as ion actuators [464] and electrolytic cells [465]. A Nafion 115-based nano-Pd loaded composite membrane has been used in electrolytic cells with a proton exchange membrane [465]. Such a membrane demonstrated 25% higher proton conductivity and reduced hydrogen permeability. An important area is the development of sensors for hydrogen [466,467,468]. Recently, hydrogen sensors based on composites containing Pd nanoparticles, nanowires, and nanosheets on various substrates have been developed [469,470,471,472,473,474]. In [475], a hydrogen sensor based a metal–organic framework with Pd nanoparticles supported on PET was proposed.

### 4.4. Water Treatment: Hydrogenation with Palladium-Containing Membranes

Water is one of the most essential resources on Earth and plays a critical role in nearly all aspects of human life, making the issue of water quality highly relevant. In recent years, numerous reviews have been published in the scientific literature addressing the assessment of surface and groundwater pollution [476,477,478,479,480,481]. The primary pollutants of groundwater include heavy metals, organic compounds (e.g., phenols and organochlorine compounds), nitrogen-containing substances, and petroleum products [482]. The extensive use of fertilizers and pesticides in agriculture significantly contributes to the pollution of surface and groundwater, highlighting the urgent need for the development of effective methods of removal of these contaminants from aqueous media [481,483]. The organochlorine compounds are among the most hazardous pollutants to human health. Of particular danger are dichloroethane, carbon tetrachloride, and trichloroethylene (with a lethal oral dose of 20–30 mL), while chloroform and tetrachloroethylene pose slightly lesser risks (a lethal oral dose of 30–80 mL).

In addition, elevated concentrations of nitrates and nitrites in drinking water have become a critical concern in recent decades. Nitrates have a harmful impact on both humans and animals, particularly through their role in forming methemoglobin in the blood, a substance that impairs oxygen transport and leads to oxygen deprivation. Excessive nitrate levels in water can cause poisoning, gastrointestinal disorders, disruptions to the excretory and endocrine systems, and the degradation of dental enamel, often resulting in the development of caries. In Europe, the maximum permissible concentrations of nitrates and nitrites in drinking water are 45 mg/L and 0.1 mg/L, respectively.

Beyond the removal of harmful pollutants from water to protect human health and the environment, the production of high-purity water is vital for advanced industries such as energy and microelectronics. One of the key challenges in these sectors is the presence of dissolved oxygen in water. In the energy industry, the dissolved oxygen content in water must be maintained at approximately 5–10 μg/L to minimize the corrosion of equipment and scale formation in heating systems, thereby extending the service life of heating networks and equipment by 10 years or more. In the semiconductor and microelectronics industries, even higher standards are required, with ultrapure water containing no more than 1 μg/L of dissolved oxygen [484].

Currently, membrane technologies are increasingly employed to address water treatment challenges due to their low energy consumption, operational flexibility, scalability, and environmental sustainability [485,486]. One promising approach for removing various waterborne contaminants involves the catalytic reduction of these substances using palladium-based catalysts and hydrogen gas as a reducing agent. This method has been extensively studied in both laboratory and pilot-scale settings [27]. Catalytic hydrogenation processes, particularly those applying palladium, are highly effective in treating contaminants such as dissolved oxygen, organochlorine compounds, nitrates, and nitrites [27,487]. Due to its high catalytic activity, the hydrogenation reactions over palladium catalysts can be conducted even at room temperature, which also enables the use of traditional polymeric porous membranes as gas–liquid membrane contactor systems.

**Figure 7:**
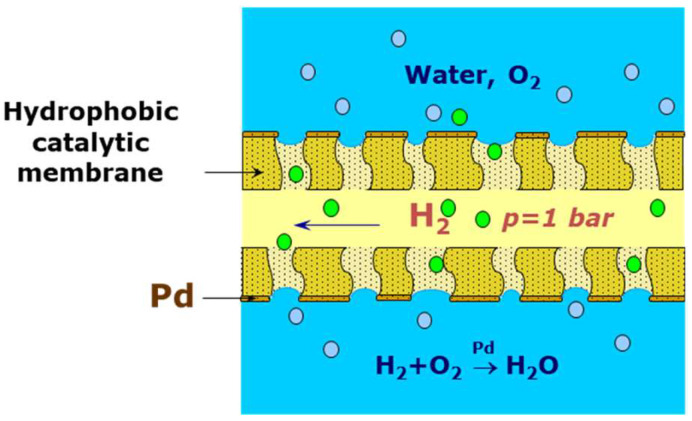
Concept of membrane reactor for hydrogenation processes in aqueous phase: example of removal of dissolved oxygen with Pd-loaded gas–liquid membrane contactor/reactor system [488].

Figure 7 illustrates the concept of such a membrane contactor/reactor system for the removal of undesired components via its hydrogenation. The membranes used in the contactor systems provide a well-defined gas–liquid interface without the mixing of two phases, and are made of (semi)hydrophobic polymers, such as polypropylene, preventing the wetting of pores with the liquid. The palladium particles are deposited on the membrane surface facing the liquid phase; therefore, the hydrogen supplied through the membrane pores dissolves in the water and shortly reacts with the substrate presented in the water once two different molecules reach the palladium catalyst. In other words, the membrane contactor/reactor ensures a more uniform supply of one of the substrates (hydrogen) to the well-defined reaction zone, which is the liquid/membrane interface.

Among different types of membranes, the hollow fiber membrane configuration enables the greatest membrane area per unit volume that maximizes the reaction zone and the increase in the interface/bulk of the liquid phase, which promotes the diffusion of the substrate and products to/from the reaction zone (membrane interface). In contrast to the more traditional two-stage process (water saturated with hydrogen is fed to the fixed-bed reactor), hydrogen is supplied at about ambient pressure to ensure its consumption only for chemical reaction and not the accumulation of excess dissolved gas in the water. The gas–liquid membrane contactors can also be used for oxygen removal by stripping it with inert gas (nitrogen) or water vapors; however, greater surface area and high purity of stripping gas are required to drop the dissolved oxygen concentration to the same level. The solubility of the target component can also be reduced by increasing water temperature up to its boiling point, but it is associated with a higher energy cost.

#### 4.4.1. Organo-Chlorinated Compounds’ Removal from Industrial and Waste Water

Catalytic hydrodechlorination using palladium is a highly effective method for treating organochlorine-contaminated groundwater and wastewater [486,489,490,491,492,493]. While earlier studies have investigated the use of other metals for catalytic water treatment [494,495,496], palladium catalysts demonstrate superior activity, stability, and selectivity for targeted processes, while also being less toxic. The capability of palladium to dissociatively absorb hydrogen enables the conversion of trichloroethylene and other organochlorine compounds into harmless substances with minimal byproduct formation, making it particularly suitable as a hydrodechlorination catalyst [27].

However, during the process of hydrodechlorination, there is a possibility of the formation of compounds that can deactivate the catalyst. For instance, hydrochloric acid, which is formed during the hydrodechlorination of trichloroethylene, can poison the catalyst. Nevertheless, [497] shows that high concentrations of H_2_CO_3_, HCO_3_⁻, CO_3_^2^⁻, SO_4_^2^⁻, and Cl⁻ do not significantly affect catalytic activity. In fact, increasing the solution pH from 4.3 to 11 results in a 30% increase in trichloroethylene conversion. Conversely, the presence of 87 mg/L SO_3_^2^⁻ or 0.4 mg/L HS⁻ rapidly deactivates the catalyst, likely due to chemisorption at the active sites.

Several strategies have been proposed to enhance catalyst stability during hydrodechlorination. The most effective methods include the addition of sodium carbonate and the use of bimetallic catalysts, such as Pd/Fe, Pd/Cu, and Pd/Au [497,498,499,500,501,502,503].

Despite these advancements, palladium-based catalysts remain the most widely used for dehydrochlorination in aqueous systems. One commonly employed catalytic reactor design involves palladium deposited on porous substrates, including aluminum oxide, silica gel, activated carbon, zeolites, or nanoparticle supports such as gold [27,490,504,505,506,507]. Substrate materials significantly influence reaction rates, as demonstrated in a study [508] where palladium supported on aluminum oxide (Pd/Al) and graphene derivatives (graphene oxide and reduced graphene oxide, Pd/GO, and Pd/rGO, respectively) are used for the hydrodechlorination of 4-chlorophenol. The study evaluated the effects of the catalyst content, initial 4-chlorophenol concentration, and solution pH on the removal process, showing that Pd/GO and Pd/rGO catalysts exhibited higher reaction rates compared to Pd/Al.

Hydrogenation reactions over a palladium catalyst can be carried out under ambient conditions. However, in conventional catalytic reactors using palladium-coated substrates, the process is conducted in two stages. First, the water is pre-saturated with hydrogen. Then, the prepared reaction mixture is introduced into a reactor containing the catalyst, where hydrodechlorination occurs as a result of hydrogen activation on the palladium surface. In contrast, catalytic membrane reactors equipped with porous Pd-containing membranes make it possible to conduct single-stage, continuous hydrodechlorination in the aqueous phase. For instance, a study [509] described the use of a catalytic membrane reactor based on a commercial palladium-coated porous ceramic tubular membrane (LikuidNanotek). In this system, water flows along the exterior of the tubular membrane while hydrogen is continuously supplied to the interior. This configuration ensures hydrogen diffusion across the membrane to the catalytically active surface, where hydrodechlorination occurs. The reactor demonstrated effective degradation of the anti-inflammatory drug diclofenac, achieving a 60% conversion rate with stable performance over 120 h in laboratory conditions.

Membrane reactors remain one of the most practical configurations for the removal of organochlorine compounds [510,511,512]. The incorporation of highly porous polymer membranes in such reactors offers several advantages, including increased catalytic surface area, enhanced device stability, modularity, and scalability. For example, commercial PVDF microfiltration membranes (Millipore, Burlington, MA, USA) impregnated with Pd/Fe catalysts and poly(methacrylic acid) (PMA) were developed for the remediation of organochlorine contaminants in groundwater. These membranes achieved a 91% reduction in trichloroethylene concentration and demonstrated that the decomposition rate of chlorine-containing compounds follows the order of carbon tetrachloride > trichloroethylene > tetrachloroethylene > chloroform [513]. Additionally, a Pd-Fe nanoparticle-integrated PMA-PVDF membrane, developed using similar polymer membranes, achieved a 92% conversion of chlorine-containing compounds. However, the catalyst required regeneration after 24 h of operation [514].

To overcome the problem of hydrogen bubble formation in the aqueous phase, it has been proposed to utilize non-porous polypropylene hollow fibers (Teijin, Ltd., Japan) coated with palladium for the removal of 1,1,1-trichloroethane (1,1,1-TCA) and trichloroethene (TCE) from water [515]. During 90 days of continuous membrane reactor operation, removal efficiencies reach up to 95% for 1,1,1-TCA and 99% for TCE. Ethane has been identified as the primary reaction product, with a selectivity of 94%. These membranes are also applied for the removal of trichloroacetic acid (TCAA) from water, a common byproduct of chlorine disinfection in wastewater containing organic matter. This problem has become especially critical during the outbreak of the pandemic coronavirus infection (COVID-19). In a membrane reactor, over 99% of trichloroacetic acid was removed from the feedwater, with acetic acid being the primary reaction product [516].

Another study [517] explores the use of a Pd-Au alloy and metallic palladium nanoparticles deposited on the surface of non-porous polypropylene hollow fiber membranes (Teijin, Ltd., Tokyo, Japan). These membrane reactors have been employed for the dechlorination of chloramphenicol. The results have shown that Pd-Au nanoparticles provided higher reaction rates and greater conversion efficiency compared to monometallic Pd nanoparticles. The complete dechlorination of chloramphenicol is achieved in the Pd-Au alloy-based membrane reactor.

Promising results have also been obtained using porous polypropylene hollow fiber membranes as catalytic substrates. For example, a study [518] implemented a one-stage water treatment process for chlorine-containing organics in a catalytic membrane reactor using palladium-coated porous polypropylene hollow fiber membranes. This approach was demonstrated using the example of the removal of trichloroethylene (TCE) from water. In this system, palladium nanoparticles were coated on the outer surface of the porous hollow fiber membranes, with hydrogen supplied to the interior of the fibers while water flowed over the outer surface. The TCE removal efficiency ranged from 96.9% to 99.6%, and the catalytic activity of the membrane remained consistently high for at least 50 h of continuous operation.

#### 4.4.2. Dissolved Oxygen Removal

Dissolved oxygen is a critical unwanted component in the ultrapure water used in the microelectronic or energy industry. Although the concentration of dissolved oxygen in water under normal conditions is relatively low (typically not exceeding 8 ppm), many industrial processes require far lower levels, often in the range of a few ppb or less. Dissolved oxygen can be removed using physical or chemical methods. While chemical methods can achieve deep purification, traditional approaches, such as reduction with sodium hydrazine hydrate or sodium sulfite at high temperatures, have significant drawbacks. These include the toxicity of hydrazine-based products and the formation of undesirable suspended solids when sodium sulfite is used [519].

Physical methods are simpler and are often employed at preliminary water purification stages or when ultra-low dissolved oxygen levels are not required [520,521,522]. However, relatively high purification efficiencies (down to 30 ppb) can be achieved using hollow-fiber membrane contactors—for example, those based on porous polypropylene hollow fibers such as Liqui-Cel industrial gas–liquid membrane contactors (Celgard X50) [484,523]. In these systems, the membrane surfaces remain unwetted by water, which is supplied to the exterior of the fibers, while a stripping gas (nitrogen, water vapor, etc.) is introduced inside the hollow fibers. The efficiency of oxygen removal increases with an increasing flow rate of stripping gas, resulting in a decrease in dissolved oxygen concentrations.

A highly effective method for the deep removal of dissolved oxygen is hydrogen reduction over a palladium catalyst, which converts oxygen into water [524,525]. One common approach to catalytic oxygen removal involves a flow-through reactor containing a nano-palladium catalyst embedded in an ion-exchange resin. This process consists of two stages: first, water is saturated with hydrogen by pressurizing it through a saturator equipped with ceramic nozzles. Then, the hydrogen-saturated water, containing dissolved oxygen, is passed through a catalyst bed, where oxygen is reduced by hydrogen. Using this method, the concentration of dissolved oxygen can be reduced to 30 ppb, but achieving this level of purification requires a minimum reaction time of 40 min [524].

An alternative approach is described in [526], where a membrane contactor composed of porous polypropylene hollow fibers is used in combination with a palladium-coated anion-exchange resin catalyst placed between the fibers. In this system, dissolved oxygen is reduced by hydrogen supplied inside the hollow fibers. An analysis revealed that the removal rate of dissolved oxygen is controlled by the liquid film formed between the catalytic particles and the external surfaces of the hollow polypropylene fibers. When the palladium content is sufficiently high, the oxygen removal rate becomes reaction-limited, and the dissolved oxygen concentration is reduced to 1.5 ppb.

In other studies [488,527,528], a one-step process for dissolved oxygen removal at room temperature without hydrogen bubbling is shown. In this approach, palladium nanoparticles are deposited on the external surfaces of porous polypropylene hollow fibers. Water containing dissolved oxygen flows along the hydrophobic, palladium-loaded exterior of the hollow fibers, while hydrogen is supplied into the fibers and diffuses through the membrane pores to the palladium-loaded surfaces. On the surface of palladium particles, hydrogen undergoes catalytic activation, enabling a heterogeneous catalytic reaction to convert dissolved oxygen into water.

When the pilot reactor is operated in the water recirculation mode (water flow rate: 25 L/h at 293 K), the dissolved oxygen concentration decreases by more than four orders of magnitude, reaching levels of 1 ppb or lower. This meets the most stringent requirements for ultrapure water in industrial applications. The activity of the catalyst formed on the hollow-fiber surfaces in the pilot catalytic membrane reactor remains stable for at least 400 h.

#### 4.4.3. Nitrate and Nitrite Removal

A variety of methods are employed for the removal of nitrates from water, including reverse osmosis, ion exchange, adsorption, electrodialysis, biological nitrification, catalytic reduction, electrocatalytic reduction, and photocatalysis. In processes such as reverse osmosis, electrodialysis, and ion exchange, a secondary step involving either concentration or neutralization is often required to complete the removal of nitrates. The catalytic conversion of nitrates to nitrogen gas is particularly effective, as it achieves high nitrate removal rates; however, in some cases, the formation of ammonia remains a challenge [476]. Three methods for the removal of nitrates from water in the membrane reactors based on non-porous polypropylene fibers are compared in [529]: using denitrifying biofilm, palladium particles, and denitrifying biofilm plated with palladium particles. The best results were obtained on a film plated with palladium particles.

The catalytic hydrogenation of nitrates is typically conducted using bimetallic catalysts comprising platinum group metals (Pt or Pd) combined with a promoter metal (e.g., Cu, Ni, Fe, Sn, In, or Ag). The catalytic reduction of nitrates proceeds through multiple stages: first, nitrates are reduced to nitrites on the promoter, and subsequently, nitrites are either reduced to nitrogen gas or converted to ammonium on palladium [530,531,532]. In 1993, Hörold et al. [530] demonstrated that both nitrates and nitrites could be reduced by hydrogen over precious metal catalysts, producing nitrogen gas and dissolved ammonia as reaction products. While nitrogen gas is desirable, the presence of ammonia in drinking water is problematic. A Pd/Cu bimetallic catalyst was proposed to reduce nitrites to nitrogen with a selectivity of 99.9%. However, although the incorporation of a second metal, such as copper, activates the catalyst for nitrate removal, it also increases ammonia formation. To address this issue, combinations of nitrate and nitrite reduction catalysts have been developed to minimize ammonia formation in drinking water to acceptable levels.

The performance of bimetallic Pd/Cu and Pd/Sn catalysts deposited on various carbon-based supports (graphite, carbon nanofibers, reduced graphene oxide, activated carbon, and carbon black) has been compared to evaluate the effect of the substrate on nitrate removal. Studies have shown that Pd-Sn catalysts deposited on carbon materials exhibit varying degrees of nitrate reduction and ammonium selectivity. Among these, Pd-Sn catalysts supported on carbon fibers demonstrated the lowest ammonium selectivity [533]. Similarly, the performance of Pd/Cu catalysts has been found to depend on the type of carbon substrate, with the highest activity observed in samples supported on carbon black [534]. The composition of the working fluid also significantly influences the reaction kinetics and product yields. Catalytic contactors based on hydrocarbon nanofibers with Pd/Cu catalysts have been investigated for nitrate reduction in potassium nitrate (KNO_3_) solutions and groundwater. The highest selectivity for nitrogen gas (79%) in deionized water was achieved at a pH of 7.0 and a hydrogen pressure of 0.1 bar [535].

The polymeric materials are also widely used in catalytic membrane reactors for nitrate and nitrite removal [536,537,538]. For instance, a study [536] demonstrates that Pd/In catalysts deposited on polymer fibers exhibit higher activity for nitrate reduction compared to inorganic supports (e.g., SiO_2_, Al_2_O_3_, carbon fibers). However, rapid catalyst deactivation is observed after just 20 min of operation. Further investigations into the stability of Pd/In catalysts deposited on polypropylene fibers revealed that the method of catalyst deposition on the membrane surface plays a critical role. Membranes with catalysts directly plated inside the membrane module exhibit the highest stability during nitrate removal [538].

## 5. Conclusions

This review provides an overview of the key properties of palladium-based alloy membranes and their potential applications in deep hydrogen purification, hydrogen production methods, fuel cells, and water purification processes for removing various impurities. Palladium membranes are considered essential tools for producing high-purity hydrogen. However, their widespread use is hindered by their high cost and relatively low production rates. To address these challenges, several strategies have been developed, including palladium alloy formation, reducing membrane thickness, surface modification, and coating thin selective layers of palladium or its alloys onto porous or non-porous substrates with high gas permeability. In addition, a promising approach is the integration of one-stage hydrogen production within palladium-containing membrane reactors, which can significantly enhance process efficiency. Progress in these technologies will be associated with the synthesis of new, highly permeable membranes containing a thin selective or protective layer of palladium alloy, including a catalytic finely dispersed coating that activates the membrane surface.

At the same time, technologies using membranes with a deposited palladium layer for the highly environmentally friendly generation of electricity in fuel cells are also being developed. In addition, the active development of synthetic approaches associated with the use of hydrogenation and dehydrogenation processes in reactors with palladium membranes, including electromembrane processes, can be expected. Some of the technologies being developed are associated with the purification of liquid or gaseous media from toxic impurities that are reduced by highly active atomic hydrogen on the surface of palladium membranes.

A particularly promising direction for future research in composite palladium-containing polymer membranes lies in the development of composite membranes based on heat-resistant polymers containing small quantities (1–5%) of palladium nanoparticles and the investigation of their gas transport characteristics (permeability to hydrogen, CO_2_, N_2_, and separation selectivity) at high temperatures for both individual gases and their mixtures. Given that the current literature describes such membrane studies in a rather fragmented manner, there is significant interest in conducting systematic research on the influence of palladium nanoparticles within the polymer matrix on the gas permeability and separation selectivity of gas pairs containing H_2_. Notably, the question of how the polymer structure affects its transport parameters upon the introduction of palladium nanoparticles remains open. Existing studies have shown varying results, with some cases demonstrating synergistic effects between the polymer and palladium, while others showed a negligible impact of palladium on gas transport characteristics.

Future work aims to identify the structural characteristics of polymers that provide enhanced separation selectivity of hydrogen-containing gas pairs when palladium nanoparticles are incorporated into the polymer matrix. This research will identify the most suitable polymers for developing highly efficient composite metal–polymer membranes for hydrogen purification and membrane catalysts for hydrogenation and dehydrogenation reactions. Heat-resistant polymers previously proposed for gas separation membranes, such as polybenzimidazoles and perfluorinated polymers, are expected to be prime candidates.

Another promising research direction involves the development of catalytic membrane reactors operating at low temperatures based on polymer membranes with catalytically active palladium particles. Recent research interest in such membrane catalysis has primarily focused on liquid-phase processes, typically applying catalysts based on transition metal nanoparticles on porous supports or homogeneous catalysts based on transition metal complexes (for hydrodehalogenation, reduction, and cross-coupling reactions). Consequently, the future development of membrane reactors based on polymer-Pd composites is likely to continue in this direction. The implementation of membrane catalysis instead of traditional methods will enable continuous, flow-through processing, while the polymer substrate will enhance catalyst durability and resistance to palladium leaching.

Furthermore, the substitution of platinum with palladium in fuel cells addresses several challenges associated with platinum usage. Specifically, palladium–platinum alloys demonstrate significantly lower adsorption of redox reaction intermediates and, consequently, higher operational stability. Importantly, the electrocatalytic activity of such elements remains unchanged. Therefore, optimizing and developing new palladium-containing electrodes for fuel cells may increase their longevity and operational stability. The key to advancing palladium-containing materials for these applications lies in optimizing both the nature of the electroactive catalyst and the polymer substrate for its deposition, enabling the regulation of catalyst activity and reduction in side reactions that occur on the catalyst surface and lead to catalyst poisoning via formed intermediates.

## Figures and Tables

**Table 1:** Comparison of the main methods of hydrogen production according to various characteristics. Average values of characteristics are estimated from [20,72,73,74].

Hydrogen Production Method	Energy Efficiency, %	Carbon Footprint, kg(CO_2_)/kg(H_2_)	Costs of Hydrogen, €/kg
Methane stream reforming	75	10	4
Methane pyrolysis	55	3	7
Biomass gasification	40	6	8
Coal gasification	70	25	1.5
Water electrolysis	70	0	6

**Table 3:** Gas transport characteristics of PBI-HFA membranes coated with continuous Pd-layer at 150 °C and 8 bar [261].

Pd Layer Thickness (nm)	Polymer Surface Treatment	Pd Coating Method ^a^	*P*, 10^−16^ mol m^−1^ s^−1^ Pa^−1^				Separation Selectivity	
			H_2_	N_2_	CO_2_	CO	H_2_/N_2_	H_2_/CO_2_
0	none	none	925	33	200	41	28	4.5
313	none	CELP	800	24	150	0	33	5.4
417	none	VELP	785	23	135	0	33.5	5.8
656	H_2_O_2_	VELP	870	0	120	0	∞	7.1
188	H_2_O_2_	VELP	780	29.5	34.5	0	27	23
130	H_2_O_2_	VELP	585	34	92	0	17	6.4
159	CO_2_ Plasma	VELP	880	21	39.5	0	41	22

^a^ CELP—conventional electroless platting, VELP—vacuum electroless platting.

**Table 4:** Examples of thermally stable polymers used for gas separation membranes.

Polymer	*T*_g_ (°C)	*T*_m_ (°C)	*T*_d_ (°C)	Reference
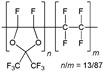 AF-2400	240	–	>360	[318]
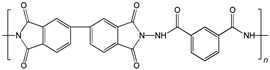	–	–	>300	[319]
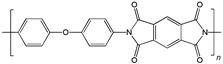	420	–	450–500	[314]
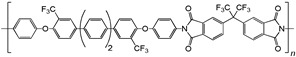	273	–	526	[311]
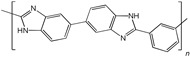 *m*-PBI	450	–	>450	[312,313]
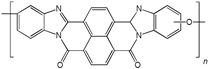	–	–	520	[294]
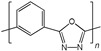	–	>400	450	[315]
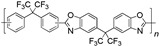	–	–	>400	[320]
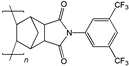	>330	–	>400	[317]
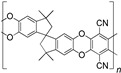	–	–	~450	[321]
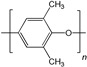	206–230	240–280	>300	[322]

**Table 6:** The performance effect of palladium introduction on composite membranes’ gas transport properties.

Membrane Material	Membrane w/o Pd			Pd-Polymer Composite			Ref.
	*P*(H_2_), 10^−16^ mol m^−1^ s^−1^ Pa^−1^	α(H_2_/CO_2_)	α(H_2_/N_2_)	*P*(H_2_), 10^−16^ mol m^−1^ s^−1^ Pa^−1^	α(H_2_/CO_2_)	α(H_2_/N_2_)	
Polysulfone + 1% Pd Polysulfone + 2% Pd Polysulfone + 3% Pd	–	4.4	6.2	–	3.7	15.2	[345]
				–	6.2	20.2	
				–	5.2	15.7	
Polycarbonate + CNT-ox ^a^ + Pd	33,000	6.1	3.3	16,000	3.9	2.2	[347]
Polycarbonate + CNT-ox ^a^ + Pd	40,000	6.4	4.1	16,000	8.0	4.2	[347]
Matrimid + ZIF-8 + Pd	150	3.3	87	230	5.1	140	[348]

^a^ Oxidized carbon nanotubes.

**Table 7:** Polymer–palladium composite-based membrane reactors for hydrogenation with hydrogen.

Membrane Material	Palladium Nanoparticles’ Introduction Method	Process	Ref.
Polyacrylonitrile, polyetherimide, and polyamidimide modified with palladium nanoclusters	Introduction of TiO_2_ into the membrane pores and further membrane treatment with a Pd(OAc)_2_ solution in methyl-ethyl ketone and subsequent NaBH_4_ reduction	Selective hydrogenation of propyne to propene	[411]
Polydimethylsiloxane with Pd nanoclusters in a polymer matrix	Introduction of Pd(OAc)_2_ solution and subsequent NaBH_4_ reduction	Hydrogenation of propyne to propene and propane	[399]
		Propene hydrogenation	[400]
Polyacrylic acid with Pd nanoparticles in a polymer matrix	Introduction of Pd(OAc)_2_ solution and subsequent NaBH_4_ reduction	Hydrogenation of propyne to propene and propane	[405]
		Cyclohexene to cyclohexane and propyne to propene and propane hydrogenation	[397]
Metathesis diblock copolymer based on norbornene with complex palladium substituent and methyltetracyclodecene	Polymerization of a monomer containing palladium in substituent and subsequent polymer treatment with hydrogen while heating	Ethylene and propene hydrogenation	[401]
		1,3-Butadiene hydrogenation	[402]
Polyamidimides with Pd nanoclusters in a polymer matrix	Introduction of Pd(OAc)_2_ solution and subsequent NaBH_4_ reduction	N_2_O hydrogenation to nitrogen	[403]
Polyvinylidene fluoride with Pd nanoclusters in a polymer matrix	Introduction of PdCl_2_ solution and subsequent NaBH_4_ reduction	Methylenecyclohexane to methylcyclohexane hydrogenation	[398]
Polyamidimide or polyether sulfone surface-modified with palladium deposited on an alumina substrate	Introduction of Pd salt solution and subsequent NaBH_4_ reduction; introduction of Pd salts solution and subsequent calcination in air	Sunflower oil hydrogenation	[396]
Phenolphthalein polyethersulfone modified with palladium nanoparticles in a polymer matrix	Injection of PdCl_2_ solution and drying at 110 °C	1-Octene hydrogenation	[410]
Cellulose acetate-based hollow fibers surface-modified with palladium nanoparticles	Hollow fibers’ immersion in PdCl_2_ solution stabilized with poly-vinylpyrrolidone and reduction with hydrazine	Selective hydrogenation of propadiene and propine to propene	[407]
		Selective hydrogenation of 1,3-butadiene to 1-butene	[408]
Cellulose acetate, polyacrylonitrile, and polysulfone-based hollow fibers surface-modified with palladium nanoparticles	Hollow fibers’ immersion in PdCl_2_ solution stabilized with polyvinylpyrrolidone and reduction with hydrazine	Hydrogenation of conjugated dienes: cyclopentadiene, 1,3-butadiene and isoprene	[404]
Hybrid membrane based on polyvinyl alcohol, ZrO_2_, and palladium nanoparticles	NaBH_4_ reduction of PdO contained in membrane	Hydrogenation of 1,5-cyclooctadiene, 3-hexyn-1-ol, 4-phenyl-3-buten-2-one, and methyl 2-acetamidoacrylate	[406]
Polyethersulfone-based hollow fibers and flat membranes surface-modified with palladium nanoparticles	Immersion of membranes in [Pd(NH_3_)_4_]Cl_2_ solution and NaBH_4_ reduction	Hydrogenation of indene, 1-dodecene, 4-isopropenyl-1-methylcyclohexene, diphenyl acetylene, nitrobenzene, and 4-nitrobenzonitrile	[409]

**Table 8:** Modification of proton-conducting membranes of methanol fuel cells with palladium for methanol permeability reduction.

Polymer Matrix	Modification Method	Impact of Pd on Methanol Permeability	Effect of Pd on Proton Conductivity	Ref.
Nafion 117	Pd impregnation	7.4x reduction	1.5x reduction	[434]
Nafion 117	Pd impregnation	1.4–1.5x reduction	1.1x reduction at low Pd content and increase at high Pd content	[435]
Nafion 117	Pd impregnation	–	1.5x reduction	[436]
Nafion 117	Pd impregnation in SC-CO_2_	15–30% reduction	10–20% reduction (depending on temperature)	[437]
Nafion 117	Pd impregnation in SC-CO_2_	1.8–7.5x reduction	Minor change or minor increase	[432]
Nafion 115	Pd/poly(1-vinylimidazole) impregnation	1.3–1.8x reduction	up to 2x reduction	[438]
Nafion	Pd impregnation in SC-CO_2_ using Pd salts with different ligands	1.2–1.7x reduction	up to 1.2x reduction	[439]
Nafion 117	Pd impregnation using an electrolytic cell	14–60% increase	5–23% increase	[440]
PETE composite with Nafion	Pd spraying (layer thickness of 20 nm)	–	5x reduction	[441]
Nafion 115 and Nafion 117	Pd spraying (layer thickness of 0.05–0.1 μm)	23–44% reduction	up to 30% reduction	[442]
Nafion 117	Pt/Pd-Ag/Pt spraying (Pd-Ag layer thickness of 0.1–1 μm)	–	–	[443]
Nafion 117	Chemical Pd coating on anode side	In 5 h methanol concentration on cathode less 2 g/L (vs. pure Nafion 117, for which it is over 10 g/L in 2 h and ramp)	–	[444]
Nafion 115	Pd spraying	1.5x reduction	–	[445]
Nafion 115	Pd-Cu spraying	1.3x reduction	–	[445]
Nafion 115	Pd spraying	1.6x reduction (with high methanol concentration)	18% increase	[446]
Nafion 115	Nano-Pd coating stabilized by 9-octadecene-1-ylamine	20% reduction	Minor increase	[447]
Nafion 115	Nano-Pd coating stabilized by trioctylphosphine	16% reduction	20–25% reduction	[448]
Nafion 112	Self-assembly of nano-Pd coating on surface	Reduction by 10+ times with 1 nano-layer and 100+ times with 5 layers	Minor reduction with 1 nano-layer and 30% reduction with 5 layers	[449]
Nafion	Nano-Pd coating stabilized by poly(diallyldimethylammonium)	10–35% reduction	Minor change	[450]
Nafion 117	Pd-SiO_2_ nanofibers introduction into polymer matrix	up to 1.5x reduction	up to 1.3x increase	[451]
Nafion 117	Pt-Pd (50–90% Pt) impregnation	–	–	[452]
Nafion 117	PLD (pulsed laser deposition) coating of Pd	More than 50% reduction	–	[453]
SPEEK ^a^	Introduction of graphite oxide nanocomposite with Pd into polymer matrix	1.2x reduction	1.2x increase	[433]
SPEEK ^a^	Introduction of graphite oxide nanocomposite with Pd grafted with L-tyrosine into a polymer matrix	1.7x reduction	1.8x increase	[433]

^a^ Sulfonated polyether ether ketone.

## Data Availability

No new data were created or analyzed in this study. Data sharing is not applicable to this article.
